# CellMarker 2.0: an updated database of manually curated cell markers in human/mouse and web tools based on scRNA-seq data

**DOI:** 10.1093/nar/gkac947

**Published:** 2022-10-27

**Authors:** Congxue Hu, Tengyue Li, Yingqi Xu, Xinxin Zhang, Feng Li, Jing Bai, Jing Chen, Wenqi Jiang, Kaiyue Yang, Qi Ou, Xia Li, Peng Wang, Yunpeng Zhang

**Affiliations:** College of Bioinformatics Science and Technology, Harbin Medical University, Harbin, Heilongjiang 150081, China; College of Bioinformatics Science and Technology, Harbin Medical University, Harbin, Heilongjiang 150081, China; College of Bioinformatics Science and Technology, Harbin Medical University, Harbin, Heilongjiang 150081, China; College of Bioinformatics Science and Technology, Harbin Medical University, Harbin, Heilongjiang 150081, China; College of Bioinformatics Science and Technology, Harbin Medical University, Harbin, Heilongjiang 150081, China; College of Bioinformatics Science and Technology, Harbin Medical University, Harbin, Heilongjiang 150081, China; College of Bioinformatics Science and Technology, Harbin Medical University, Harbin, Heilongjiang 150081, China; College of Bioinformatics Science and Technology, Harbin Medical University, Harbin, Heilongjiang 150081, China; College of Bioinformatics Science and Technology, Harbin Medical University, Harbin, Heilongjiang 150081, China; College of Bioinformatics Science and Technology, Harbin Medical University, Harbin, Heilongjiang 150081, China; College of Bioinformatics Science and Technology, Harbin Medical University, Harbin, Heilongjiang 150081, China; College of Bioinformatics Science and Technology, Harbin Medical University, Harbin, Heilongjiang 150081, China; College of Bioinformatics Science and Technology, Harbin Medical University, Harbin, Heilongjiang 150081, China

## Abstract

CellMarker 2.0 (http://bio-bigdata.hrbmu.edu.cn/CellMarker or http://117.50.127.228/CellMarker/) is an updated database that provides a manually curated collection of experimentally supported markers of various cell types in different tissues of human and mouse. In addition, web tools for analyzing single cell sequencing data are described. We have updated CellMarker 2.0 with more data and several new features, including (i) Appending 36 300 tissue-cell type-maker entries, 474 tissues, 1901 cell types and 4566 markers over the previous version. The current release recruits 26 915 cell markers, 2578 cell types and 656 tissues, resulting in a total of 83 361 tissue-cell type-maker entries. (ii) There is new marker information from 48 sequencing technology sources, including 10X Chromium, Smart-Seq2 and Drop-seq, etc. (iii) Adding 29 types of cell markers, including protein-coding gene lncRNA and processed pseudogene, etc. Additionally, six flexible web tools, including cell annotation, cell clustering, cell malignancy, cell differentiation, cell feature and cell communication, were developed to analysis and visualization of single cell sequencing data. CellMarker 2.0 is a valuable resource for exploring markers of various cell types in different tissues of human and mouse.

## INTRODUCTION

The development of single-cell sequencing technology provides powerful technical support for studying the gene structure and gene expression status of cells and exploring the heterogeneity between cells from the level of single cell (1,2). Moreover, single-cell RNA sequencing (scRNA‐seq) is a reliable tool for analyzing cell heterogeneity (3,4). In recent years, a large number of studies have been carried out based on single-cell RNA sequencing (scRNA-seq) data, including the exploration of intratumoral heterogeneity and the cross-linking between cells and tumor microenvironment (TME) in a variety of cancers (5–7). One of the most widespread and remarkable applications is to dissect complex cellular heterogeneity and construct comprehensive maps of all cell types in different tissues or organs (8,9). With the advent of high-throughput and large-scale single-cell sequencing technology, markers of different cell types have been gradually disclosed (10,11). In order to distinguish different cell types in different tissues, we reported the first version of CellMarker database (CellMarker 1.0), which enabled users to search for all known experimentally supported markers for different cell types in various organs in humans or mouse.

With the increasing interest in research at the single-cell level and the application of high-throughput techniques, the number of markers in various cell types has increased rapidly (12). In addition, intratumoral heterogeneity, intercellular communication and cell differentiation trajectories based on single-cell data have also been extensively studied (13–15). For example, Zhou *et al.* demonstrated the intratumoral heterogeneity of osteosarcoma cells and their TME in osteosarcoma tissues (16). Zhang *et al.* used single cell transcriptomic datasets to dissect cellular diversity and intercellular crosstalk of human ICCs (13). In addition, Li *et al.* identified the limbal stem cell population and uncovers novel cell types mapping the differentiation trajectory in heterogenous limbal basal epithelium (17). However, these analyses are based on annotating the right cell type. Therefore, it is urgent to update CellMarker with more resources and improved tools. Notably, cell markers can be divided into different groups based on single-cell sequencing technologies, including 10x Chromium, Smart-seq2, and Drop-seq, etc. In recent, some databases manually recruit marker genes for different cell types from available literature information, such as PanglaoDB (18), PCMDB (19) and CancerSEA (20). These databases provide valuable resources for annotations of cell clusters. However, the information stored in these databases has certain limitations. The tissue source, type and sequencing technology of markers are lacking. Determining the tissue source can improve the accuracy of cell annotation. However, there is still a lack of a global, high-quality database storing and classifying markers of different cell types in various human and mouse tissues.

In recent years, advances in tissue isolation and high-throughput sequencing at the single-cell level have enabled the generation of single-cell RNA-sequencing (scRNA-seq) datasets, which are increasingly entering the public domain (21). The large amount of single-cell sequencing data has created new opportunities to study the tumor microenvironment, cellular heterogeneity, molecular mechanisms of disease, and more (22). The development of fast, customizable single-cell data analysis and visualization tools can help users quickly analyze data, achieve cell annotation, analyze cell differentiation trajectories, identify malignant cells, analyze cell-to-cell communication, etc., so as to utilize existing single-cell sequencing data resources for research tumor heterogeneity, dissecting disease analysis mechanisms, identifying disease and prognostic biomarkers.

To meet these needs, we updated CellMarker 1.0 to version 2.0 (CellMarker 2.0), which added 36 300 tissue-cell type-marker entries by reviewing 24 591 published papers. This is an increase of 474 tissues, 1901 cell types and 4566 markers over the previous version. CellMarker 2.0 provides experimentally supported markers for various cell types in different tissues of humans and mouse, including tissue source, sequencing technology, marker type and other information. In addition, we developed six interactive Web tool platforms for single cell sequencing transcriptome data, including cell annotation, cell clustering, cell malignancy, cell differentiation, cell feature and cell communication. We hope that CellMarker 2.0 will become an important resource for researchers to annotate cells. All the information about CellMarker 2.0 is available free at http://bio-bigdata.hrbmu.edu.cn/CellMarker or http://117.50.127.228/CellMarker/.

## IMPROVED EXPANSION AND NEW FEATURES

### Data expansion and pre-processing

**Figure 1.**
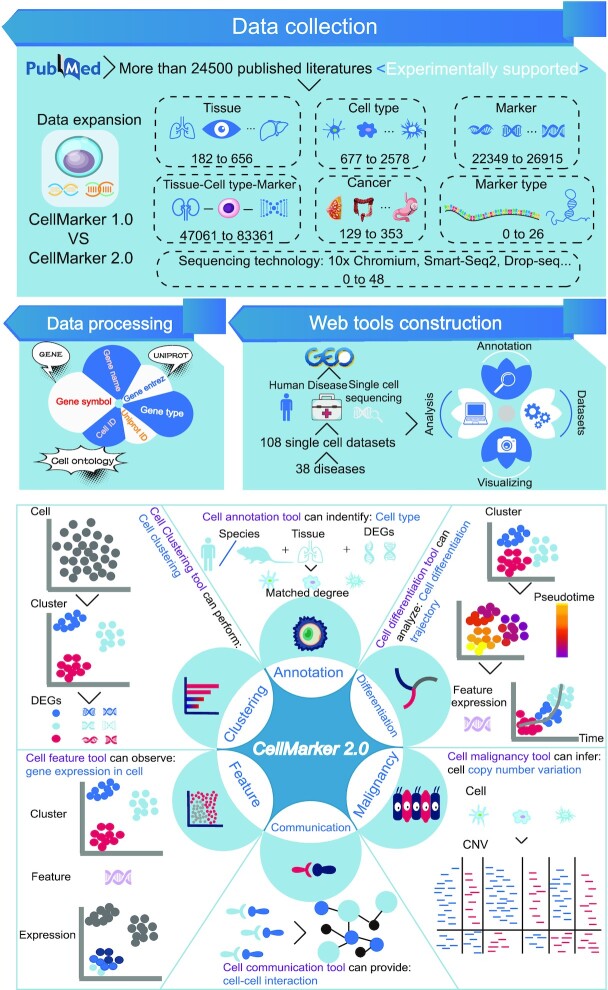
Data expansion and features of CellMarker 2.0. The upper panel is the database content, which includes experimentally validated markers for different cell types of various tissues in humans and mouse, and the construction of web tools. The lower panel shows the six functional modules of the new web tool in CellMarker 2.0, which provide a flexible way to analyze single-cell transcriptome data.

The updated CellMarker 2.0 contains more markers for various cell types in different tissues of human and mouse (Figure 1 and Table 1). First, we screened about 102 000 studies (mainly from 2019 to 2022) in the PubMed database that, in addition to using a similar combination of keywords as CellMarker1.0 (including ‘single cell sequencing’, ‘single cell RNA sequencing’, ‘single cell RNAseq’, ‘ScRNAseq’, ‘identify cell marker(s)’, ‘identify surface marker(s)’, ‘identify cell specific marker(s)’, ‘identify cell signature(s)’, ‘identify cellular signature(s)’, ‘identify surface signature(s)’ and ‘identify cell specific signature(s)’), added keyword like ‘classify cell type’. Furthermore, we further screened journal articles with impact factors >7, and finally got 24 591 studies.

**Table 1.** Comparison of the data included in CellMarker 2.0 and CellMarker 1.0

Feature	CellMarker 1.0	CellMarker 2.0	Fold increase
Tissue-cell type-marker	47 061	83 361	1.77
Tissue	182	656	3.60
Cell type	677	2578	3.81
Marker	22 349	26 915	1.21
Cancer type	129	353	2.52
Sequencing technologies	-	48	New
Marker type	-	26	New
Single cell web tools	-	108 datasets	New

We subsequently extracted markers for different cell types in various organs in humans or mouse, which were confirmed by strong source, including experiment, review and different sequencing technologies. If markers for various cell types in human or mouse are verified, the information is extracted. In addition, we also extracted some marker information of tumor cells, including different cancer subtypes. The related information of species and tissue type were also recorded in detail. Methods and principles for collecting data can be found in CellMarker 1.0. Compared with the previous version, we have added 224 cancer types, and CellMarker 2.0 currently contains 355 cancer types-related cell markers. Among them, there are 278 cancer types in human and 94 cancer types in mouse.

In addition, we have added more detailed information to more fully characterize markers for various cell types in different species, including marker type, gene symbol and entrez ID from Gene database (http://www.ncbi.nlm.nih.gov/gene), gene name and protein ID as well as UniProt database (23). Moreover, the tissue type ID and cell type ID are from Uberon muti-species anatomy ontology(24) and Cell Ontology(25), respectively. CellMarker 2.0 includes 52 987 tissue-cell type-marker association entries among 429 tissues, 1715 cell types and 16 679 markers in the human. CellMarker 2.0 also contains 32 285 tissue-cell type-marker association entries among 399 tissues, 1434 cell types and 12 504 markers in the mouse.

### Single cell sequencing technologies of cell markers

Single-cell RNA sequencing (scRNA-seq) enables whole-transcriptome profiling of single cell, revealing exciting biological and medical insights and providing new possibilities for solving biological and medical questions (26). In recent years, with the development of single-cell sequencing, many single-cell sequencing technologies have emerged, giving us the ability to classify cell markers into different groups according to the sequencing technology. CellMarker 2.0 contains cell markers derived from 48 sequencing technologies, including 10× Chromium, Smart-seq2 and Drop-seq, etc.

### Gene types of cell markers

In recent years, with the rapid development of biotechnology, tens of thousands of genes have been gradually discovered, and the role of genes has been gradually revealed, such as coding genes and non-coding genes. However, classifying genes can help better understand their function. At present, there are many public databases that record the classification of genes, such as Gene (http://www.ncbi.nlm.nih.gov/gene) and GENCODE(27). This allowed us to characterize the types of cellular markers. CellMarker 2.0 contains 26 different types of markers, including protein-coding genes, lncRNAs, microRNAs, etc.

### Newly integrated scRNA-seq web tool for analyzing single cell transcriptome data

With the rapid expansion of the available expression profiles obtained by high-throughput sequencing technology at the single-cell level, it is critical to integrate an efficient single cell analysis tool for analyzing single-cell datasets. In CellMarker 2.0, we integrated a single cell analysis and visualization tool that allows for custom analysis based on the provided single cell dataset. First, we searched the Gene Expression Omnibus (28) (GEO: https://www.ncbi.nlm.nih.gov/geo/) using the keyword ‘single-cell sequencing’, then we limited the species to ‘human’ and manually recruited the single-cell transcriptome data of major human diseases, and finally obtained 108 sets of single-cell data sets, including 38 diseases (Alzheimer's disease, Atherosclerosis, Triple negative breast cancer, dementia with Lewy bodies, gastric cancer, gastrointestinal neuroendocrine cancer, meningioma, Parkinsons disease, acute myeloid leukemia, breast cancer, COVID-19, diabetes, heart failure, hypoplastic left heart syndrome, relapsing-remitting multiple sclerosis, human pleuropulmonary blastoma, lung cancer, non-small cell lung cancer, prostate cancer, small cell lung cancer, melanoma, glioma, HIV, bladder cancer, cervical cancer, colorectal cancer, coronary atherosclerotic heart disease and dilated cardiomyopathy, head and neck squamous cell carcinoma, pancreatic adenosquamous carcinoma, pancreatic ductal adenocarcinoma, pancreatic neuroendocrine tumor, papillary thyroid carcinoma, abdominal aortic aneurysm, clear cell renal cell carcinoma, liver cancer, ovarian cancer, hepatocellular carcinoma and renal tumor) and 1 467 748 cells. The single cell web tool is equipped with six key functions (Figure 1). The Cell annotation module allows users to define cell types by selecting species, tissues and input genes based on marker information stored in CellMarker 2.0. Cell clustering module allows users to perform cluster analysis on single-cell transcriptome data based on UMAP and t-SNE dimensionality reduction methods, and to obtain differentially expressed gene between different clusters at different resolutions. This function module is implemented based on the R package Seurat(29). Cell malignancy allows users to obtain the copy number variation of malignant cells in different datasets. This functional module is implemented based on the R package InferCNV (v1.12.0) (30). Cell differentiation allows users to obtain cell differentiation trajectories of different datasets and the expression changes of interesting genes over time. This functional module is implemented based on the R package Monocle 3 (31). The Cell feature allows users to obtain the expression of feature genes in different clusters based on UMAP and t-SNE dimensionality reduction methods. This function module is also implemented based on the R package Seurat (v4.0)(29). Cell communication allows users to perform cell communication analysis of single-cell transcriptome data based on ligand receptors. This functional module is implemented based on CellPhoneDB (v3)(32). The identification of cell types for single cell data provided in all functional modules is based on marker information recorded in CellMarker 2.0.

## DATABASE CONSTRUCTION AND IMPROVED USER INTERFACE

CellMarker 2.0, performed data management using MySQL software (v5.5). The web pages were developed using Java server pages and deployed on the Tomcat web server (v6). Several Java script plugins such as jQuery (v1.11.3), Datatable (1.10.10), and ECharts (v5.0) were used for data table creation and visualization. All statistical analyses were performed using R framework (v4.1.0). The CellMarker 2.0 database is freely available at http://bio-bigdata.hrbmu.edu.cn/CellMarker or http://117.50.127.228/CellMarker/. The version 1.0 of CellMarker, also remains available. To access CellMarker 1.0, users can visit links from the CellMarker 2.0, homepage, or directly at http://bio-bigdata.hrbmu.edu.cn/CellMarker1.0.

**Figure 2.**
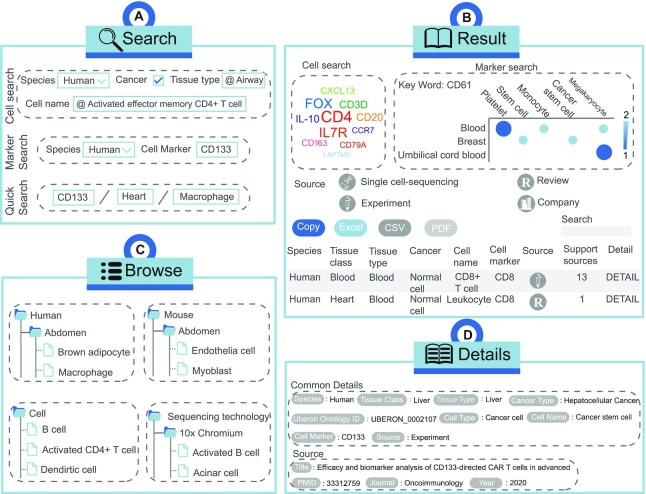
Workflow and case study of basic functions of CellMarker 2.0. (**A**) The interface of the cell search, marker search and advanced search modules using human-airway-activated effector memory CD4+ T cell, human-CD133, CD133/heart/macrophage as examples. (**B**) The interface of the browse module including human, mouse, cell and sequencing technology. (**C**) Query results for CD8 in human. **(D)** Common details and source for CD133 in human.

**Figure 3.**
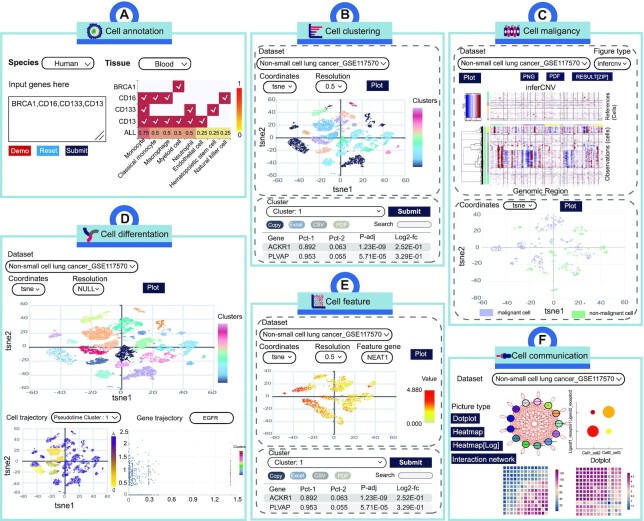
Workflow and case study using web tools in CellMarker 2.0. (**A**) Cell annotation tool can identify cell types. (**B**) Cell clustering tool includes clustering and differential expression analysis. (**C**) Cell malignancy tool can infer copy number variation of cells. (**D**) Cell differentiation tool can analyze the differentiation trajectories of cells. (**E**) Cell feature tool can observe gene expression in cells and differential expression analysis. (**F**) Cell communication tool can perform cell-cell interaction analysis.

CellMarker 2.0 shows a friendly interface and provides flexible data access route that allow users to query the database in just a few steps. (i) On the ‘HOME’ page, a fast search engine is available for users to directly investigate data. The user can search by tissue type, cell name and cell marker. All possible records are displayed on the search results page. To obtain records of interest, users have the flexibility to reorder the result table by clicking on the headings of different columns or to filter the results twice through the search box. The last column takes the user to the details page indicating gene symbols, gene IDs, gene names, protein IDs, publication information (i.e. title, PubMed ID, journal and publication year) and the cross references to external databases (Figure 2A, B, D). (ii) The ‘Search’ page provides ‘cell search’, ’marker search’ and ‘quick search’. In cell search, users can get a more detailed and systematic search by limiting to descriptions of species, tissues, and cell types of interest. In the search page of Marker search, users can search by gene alias, gene symbol and gene ID. In the quick search interface, users can search by entering tissue, cell type or cell marker (Figure 2A). (iii) CellMarker 2.0 also provides a browse page to access the dataset based on different classifications (Figure 2C). (iv) The ‘Cell Tools’ contains six functional modules that allow users to use them and the provided single-cell transcriptome data for single-cell analysis and visualization. (a) From the ‘Cell annotation’ page, user can obtain the cell type score and matching of cell marker by limiting species and tissues and inputting differentially expressed genes to identify cell types (Figure 3A). (b) From the ‘Cell clustering’ page, users can utilize interactive and customizable functions, including cell clustering based on different resolution or dimensionality reduction and differential expression analysis (Figure 3B). (c) From the ‘Cell malignancy’ page, users can customize parameters to get copy number variation of cells and visualize malignant and non-malignant cells in the different single cell datasets (Figure 3C). (d) From the ‘Cell differentation’ page, users can perform complex functions, including cell clustering, cell differentiation trajectory and gene trajectory (Figure 3D). (e) From the ‘Cell feature’ page, cell clustering and differential expression analysis are also provided, and more importantly, users can input feature genes to see their expression in different clusters (Figure 3E). (f) From the ‘Cell communication’ page, users can obtain detailed information on cell-cell interactions, including the interaction network, ligand-receptor expression between cells, and the number of ligand-receptor interactions between cells (Figure 3F). (v) CellMarker 2.0 is a totally open resource, and users can obtain all data from the ‘Download’ page. (vi) In ‘Help’ page, users can get a detailed tutorial about how to use CellMarker 2.0.

## CONCLUSIONS AND FUTURE EXTENSIONS

In the first version of the CellMarker database, CellMarker 1.0, only a limited number of cell types and markers were found in humans and mouse. With the development of high-throughput sequencing technology, the number of cell markers has increased significantly in recent years. The rapid growth of related literature indicates the urgent need to collect corresponding data sets and update the first edition of CellMarker database. At present, the data set and function of CellMarker 2.0 have been greatly improved. CellMarker 2.0 expanded to 656 tissues, 2578 cell types and 26 915 cell markers. With the development of single-cell sequencing technology and the continuous progress of biotechnology, we are able to classify cell markers in terms of sequencing technology and gene type. Such classification is very valuable for further understanding the role of cellular markers. Notably, single-cell analysis tools will fill the gap between the availability of single-cell transcriptome data and the delivery of comprehensive information to users, thus facilitating further investigation by investigators. Firstly, more and more cell markers of different cells (such as some new/rare cell types) will be identified by single-cell RNA sequencing technology (33), therefore, we will continue to follow single-cell sequencing studies and update the database by frequently adding new cell markers. Secondly, cell markers of other species, such as *Zebrafish*, *Drosophila melanogaster* and *Caenorhabditis elegans*, will be added in future versions of CellMarker to provide users with more comprehensive CellMarker information. Finally, we will continue to maintain and update the CellMarker database with additional datasets and web tools.

## DATA AVAILABILITY

All the data used in the analysis can be obtained at http://bio-bigdata.hrbmu.edu.cn/CellMarker/.
